# A type III complement factor D deficiency: Structural insights for inhibition of the alternative pathway

**DOI:** 10.1016/j.jaci.2018.01.048

**Published:** 2018-07

**Authors:** Christopher C.T. Sng, Sorcha O'Byrne, Daniil M. Prigozhin, Matthias R. Bauer, Jennifer C. Harvey, Michelle Ruhle, Ben G. Challis, Sara Lear, Lee D. Roberts, Sarita Workman, Tobias Janowitz, Lukasz Magiera, Rainer Doffinger, Matthew S. Buckland, Duncan J. Jodrell, Robert K. Semple, Timothy J. Wilson, Yorgo Modis, James E.D. Thaventhiran

**Affiliations:** a Cancer Research UK Cambridge Institute, Cambridge, United Kingdom; b Department of Clinical Immunology, Cambridge University Hospitals National Health Service Trust, Addenbrooke's Hospital, Cambridge, United Kingdom; c Molecular Immunity Unit, Department of Medicine, Medical Research Council (MRC) Laboratory of Molecular Biology, Cambridge, United Kingdom; d Division of Structural Studies, MRC Laboratory of Molecular Biology, Cambridge, United Kingdom; e Department of Immunology, Royal Free London National Health Service Foundation Trust, London, United Kingdom; f Walter and Eliza Hall Institute of Medical Research, Parkville, Australia; g Wellcome Trust–MRC Institute of Metabolic Science, Addenbrooke's Hospital, Cambridge, United Kingdom; k Department of Medicine, University of Cambridge, Addenbrooke's Hospital, Cambridge, United Kingdom; h Leeds Institute of Cardiovascular and Metabolic Medicine, Leeds Institute of Genetics, Health and Therapeutics (LIGHT) Laboratories, University of Leeds, Leeds, United Kingdom; i University of Edinburgh Centre for Cardiovascular Sciences, Queen's Medical Research Institute, Little France Crescent, Edinburgh, United Kingdom; j Department of Microbiology, Miami University, Oxford, Ohio; l MRC Toxicology Unit, University of Leicester, Leicester, United Kingdom

To the Editor:

We investigated an alternative complement pathway (AP) deficiency in a patient with absent alternative pathway hemolytic activity but normal classical pathway hemolytic activity recovering from invasive meningococcal infection (for patient and sibling details, see Patient details in this article's Online Repository at www.jacionline.org). Serum reconstitution with proximal AP components suggested a factor D (FD) deficiency (Fig 1, *A*). Sanger sequencing of *CFD* identified a rare homozygous missense mutation (c.602G>C) in exon 4 in the patient (II-1) and sibling (II-2), resulting in an arginine to proline substitution (p. R176P) (see Fig E1, *A*, and reference E9 in this article's Online Repository at www.jacionline.org). This genotype cosegregated with an alternative pathway hemolytic activity–null phenotype, as the parents, both heterozygotes, had normal alternative pathway hemolytic activity (Fig 1, *B*). In contrast to previously confirmed FD deficiencies,^1^, ^2^, ^3^ all members of the pedigree had normal levels of circulating FD, as corroborated by Western blot (see Fig E1, *B*). Meanwhile, identical circular dichroism spectra and melting curves of recombinant wild-type (WT) and R176P FD precluded gross changes in FD structure or stability, suggesting a functional deficiency (Fig 1, *C*, and see Fig E1, *C*). We assessed the cleavage of C3b-bound factor B (FB) by recombinant WT and mutant FD (R176P, R176A, R176Q). WT FD could cleave C3b-bound FB to produce fragments Bb and Ba. Conversely, R176P FD demonstrated diminished *in vitro* catalytic activity at all concentrations and had negligible activity at physiological concentration (0.04 μmol/L) (Fig 1, *D*, and see Fig E1, *D*). Reconstitution of FD-depleted serum with R176P FD also demonstrated impaired AP-mediated hemolysis (see Fig E1, *E*).

**Fig 1:**
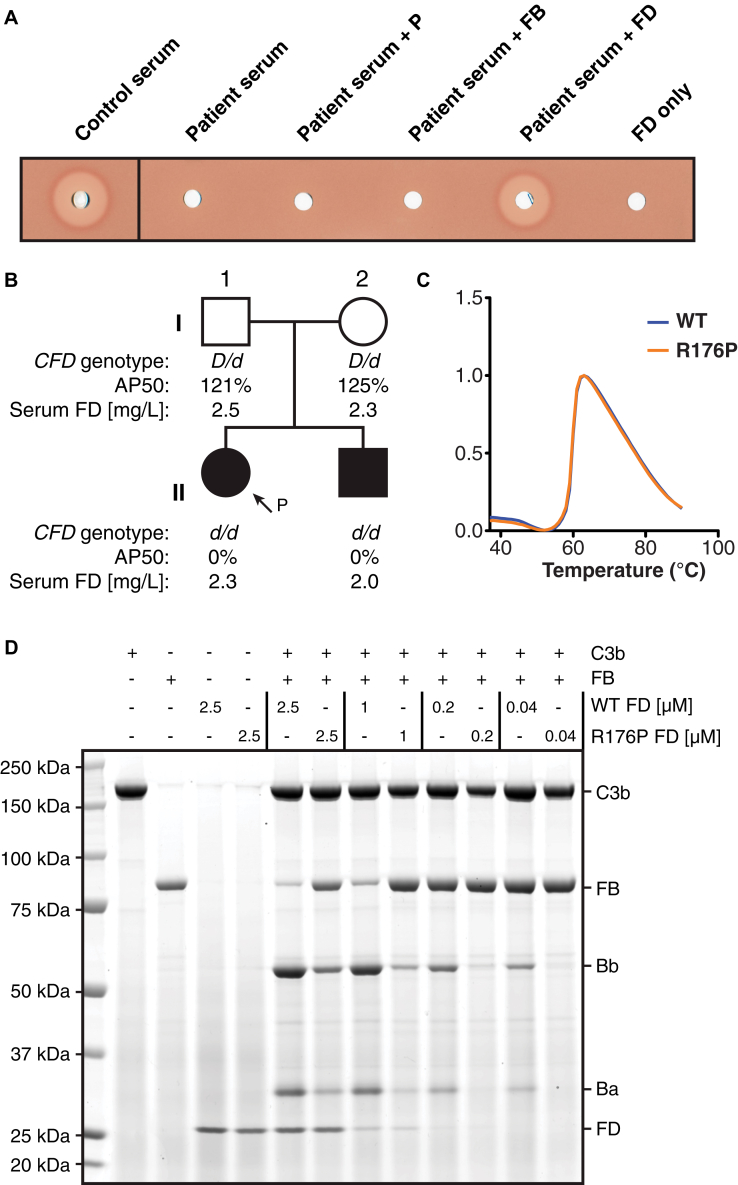
Assessing the contribution of mutation R176P to AP dysfunction. **A,** Alternative pathway hemolytic activity (AP50) assay assessing patient serum supplemented with properdin (P), FB, or FD. **B,** The immediate family pedigree of the patient with the *CFD* genotype, serum AP50, and serum FD concentrations displayed. *D* represents the WT allele and *d*, the mutant allele (c.602G>C). **C,** Thermal shift assay of WT and R176P FD. **D,** Serial dilutions of recombinant WT or R176P FD were incubated with C3b and FB. The SDS-PAGE gel, stained with AcquaStain, shows the individual proteins and resultant products.

FD's serine protease activity depends on obligatory binding to the C3bB complex via 4 exosite loops (residues 132-135, 155-159, 173-176, 203-209). This leads to rearrangement of the self-inhibitory loop (199-202), allowing realignment of His41 and Asp89 with Ser183 to form the active catalytic triad (see Fig E2, *A* and *B*, in this article's Online Repository at www.jacionline.org).^4^, ^5^ Mutation R176P lies outside the active site, within one of the FB-binding exosite loops. We used molecular dynamics (MD) stimulations to study how the R176P mutation affects the FD protein fold (see Fig E2, *C*). In mutant FD, we observed a rearrangement of the exosite loop 155-161 within 50 nanoseconds of simulation (Fig 2, *A*). This was unexpected because loop 155-161 was not in direct contact with residue 176. Average structures generated from the final 50 nanoseconds of simulation for WT and mutant FD (R176P and R176A) demonstrated that key FB-binding residues Asp161 and Arg157 were shifted by 4.3 Å and 1.9 Å, respectively (Cα average position) (Fig 2, *B*). Superimposing these MD average structures onto the crystal structure of the C3bB-D complex revealed that Asp161 and Arg157 assumed a conformation that no longer supported binding due to loss of shape and charge complementarity to the FB surface (Fig 2, *C*). The other 3 exosite loops retained their binding-competent conformations. After assuming the new conformation, exosite loop 155-161 demonstrated higher conformational mobility (root mean square fluctuation) relative to WT (Fig E2, *D* and *E*). In contrast, the mobility of loops containing catalytic residues His41 and Asp89 decreased in the mutants. Using the distance between His41 and Ser183 during MD simulations as a proxy for the active site conformation, we observed that WT could sample the short distance necessary for a catalytically active conformation (Fig 2, *D*). Conversely, in both mutant simulations, the distance remained larger, consistent with His41 pointing away from the active site. Therefore, in addition to disruption of key FB-binding residues, mutations R176P and R176A appear to stabilize the self-inhibited conformation of free FD.

**Fig 2:**
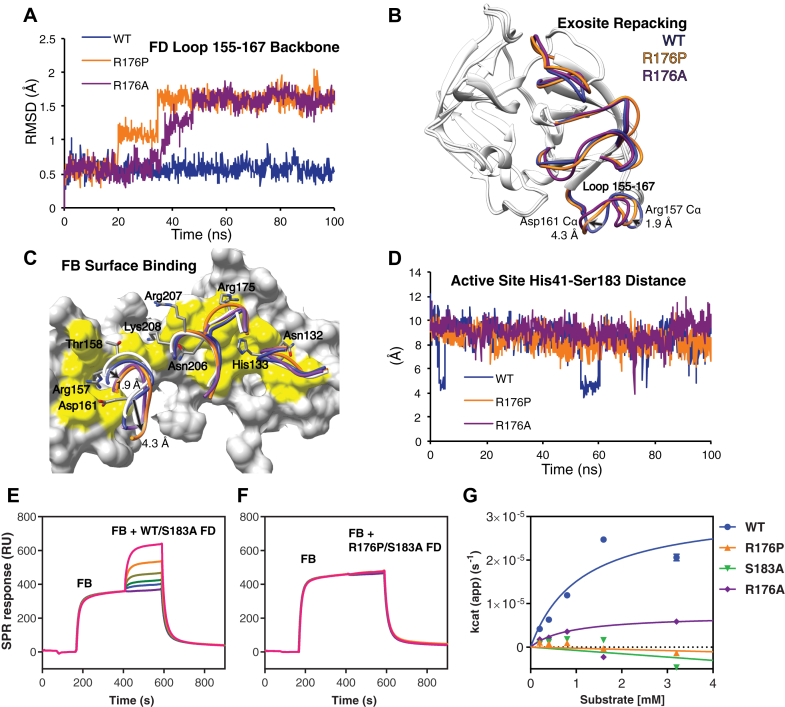
Defining the effects of the R176P mutation on FD function. **A** and **B,** FB-binding exosite loop 155-167 assumes a new conformation in mutant FD simulation. *Arrows* highlight average Cα position shifts of 2 residues that bind C3bB in the R176P FD simulation. **C,** Loss of shape complementarity at the FD-C3bB interface. FD exosite loops from published cocrystal structures (*white*, Protein Data Bank ID: 2XWB) overlaid with the simulated loops of WT and mutant FD. **D,** Distance sampled between the active site Nε2 nitrogen of His41 and Oγ of Ser183 during each simulation. The shorter distance is necessary for catalytic activity. **E** and **F,** Surface plasmon resonance binding measurement of enzymatically inactive recombinant FD (R176P/S183A or WT/S183A) to C3bB complex. **G,** Steady state kinetics for Z-Lys-SBzl cleavage by WT, R176P, R176A, and catalytically inactive control S183A FD. *RMSD*, Root-mean-square deviation.

To assess the binding of FD to C3bB, we used surface plasmon resonance. Coinjection of catalytically inactive FD (WT/S183A) with FB demonstrated a dose-dependent increase in binding to C3b and complex formation (Fig 2, *E*). In contrast, R176P/S183A FD lacked any detectable binding (Fig 2, *F*). Consistent with the stochastic transitions of free WT FD to the active conformation observed in the MD simulation, FD has a low level of esterolytic activity toward a small synthetic substrate, Z-Lys-SBzl (Fig 2, *D*). Surprisingly, R176P FD demonstrated a loss of esterolytic activity similar to the active site mutant, S183A (see Fig 2, *G*).

Deficiency of properdin, the most common AP deficiency, can result from absent (type I), low (type II), or normal but nonfunctioning (type III) protein levels (for reference, see reference E10 in this article's Online Repository at www.jacionline.org). Meanwhile, previously confirmed deficiencies of activating complement serine proteases have all resulted in low or absent gene product. We have identified a unique deficiency: R176P FD is fully expressed and stable, but enzymatically inert, constituting a functional or type III deficiency. Recent preclinical evidence^6^ that FD-deficient mice are susceptible to diabetes prompted metabolic assessment in the FD-deficient patients. No abnormality was detected (for details, see Functional FD deficiency does not result in impaired oral glucose tolerance section, Fig E3, and Table E1 in this article's Online Repository at www.jacionline.org).

Overactivation of AP is implicated in numerous inflammatory disorders, including age-related macular degeneration. Therefore, blockade of the AP by targeting the rate-limiting enzyme, FD, is an attractive approach to controlling disease progression. An anti-FD Fab fragment targeting the 2 distal exosite loops has shown some benefit in phase II clinical trials for treatment of dry age-related macular degeneration.^7^ *In vitro* studies indicate that it inhibits binding to the C3bB complex but increases esterolytic activity toward small-molecule substrates.^8^ This may result in unwanted clinical effects due to nonspecific activity or limit its efficacy *in vivo*. In the case of R176P FD, both FB-binding and esterolytic activity are abrogated through exosite hindrance and stabilization of the self-inhibited state. Loop 173-176 is thus a promising target for allosteric inhibitors of FD that stabilize the inhibitory loop in addition to binding-blockade. A structure-based design approach to targeting FD has recently succeeded in identifying candidate FD inhibitors where high-throughput screens had failed,^9^ highlighting the benefits of integrating structural information into candidate drug screens. Comprehensive definition of the structural and molecular determinants of *in vivo* FD activity is critical for this. This study of the R176P mutation demonstrates how in-depth mechanistic analysis of rare complement deficiencies can deliver such insight validated clinically by *in vivo* human evidence of AP blockade.

Our acknowledgments can be found in this article's Online Repository (at www.jacionline.org).

## Figures and Tables

**Fig E1:**
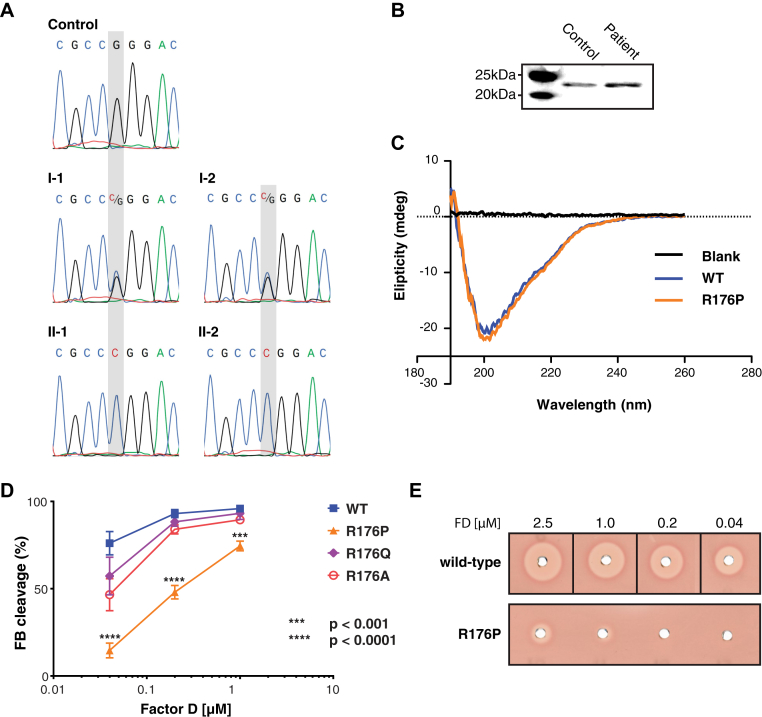
Mutation R176P results in a type III FD deficiency. **A,** Chromatograms for the DNA sequence adjacent to position c.602 are shown for each member of the pedigree. The identified variant is rare: the Exome Aggregation Consortium's ExAC database reports mutation R176P (variant 19:861943 G/C) at an allele frequency of 1.049 × 10^−4^, with no homozygotes.^E9^**B,** Western blot analysis of FD in serum from the patient and healthy control. **C,** Secondary structural compositions of WT and R176P FD were evaluated using circular dichroism spectroscopy. **D,** Comparison of *in vitro* catalytic activity of recombinant WT, R176P, R176Q, and R176A FD in terms of FB cleavage. ***P < .001; ****P < .0001. **E,** Recombinant WT and R176P FD were tested for the ability to reconstitute AP50 when added to FD-depleted serum.

**Fig E2:**
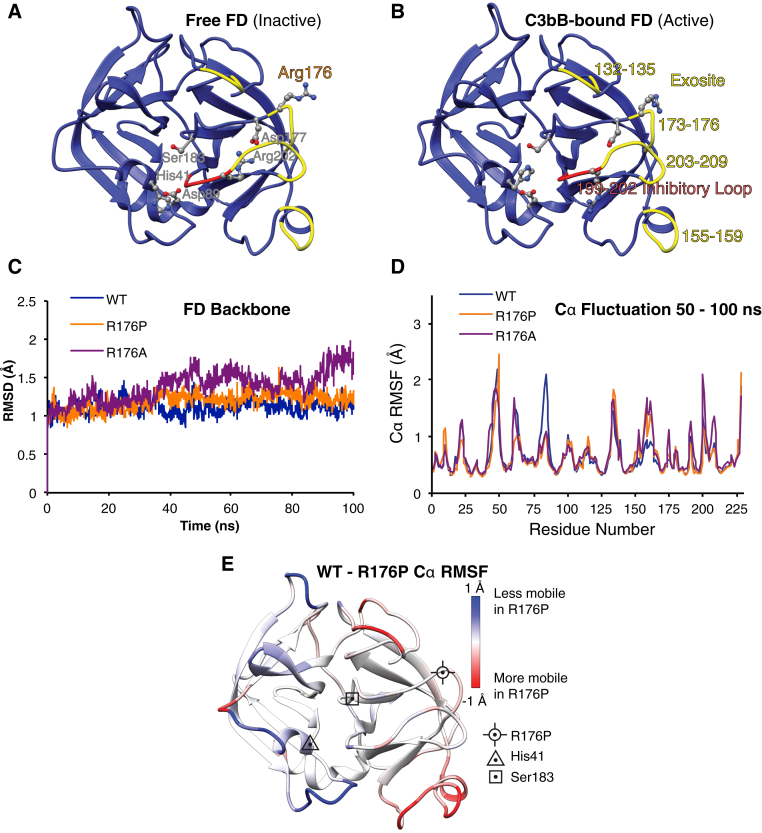
Mutation R176P stabilizes the self-inhibited state of FD. **A,** Structure of free FD^E3^ (PDB ID: 2XW9) showing the catalytic triad (Ser183-His41-Asp89) in an inactive conformation stabilized by the self-inhibitory loop 199-202 (*red*) and an ion bridge between Asp177 and Agr202. The exosite loops are shown in *yellow*. **B,** Structure of C3bB-bound FD^E3^ (PDB ID: 2XWB) omitting the C3b and FB components. FD exosite loops retain a conformation similar to that of unbound FD. **C,** WT, R176P, and R176A structures were stable over 100 nanoseconds of unrestrained molecular dynamics simulation with explicit solvent. **D,** Root mean square fluctuation (*RMSF*) in WT and mutant FD over the second half of the trajectory. **E,** Differences in WT versus R176 RMSF mapped to the FD structured. MD predicted increased mobility in exosite loops, notably 155-167, and decreased mobility in loops carrying the catalytic His41 and Asp89 residues.

**Fig E3:**
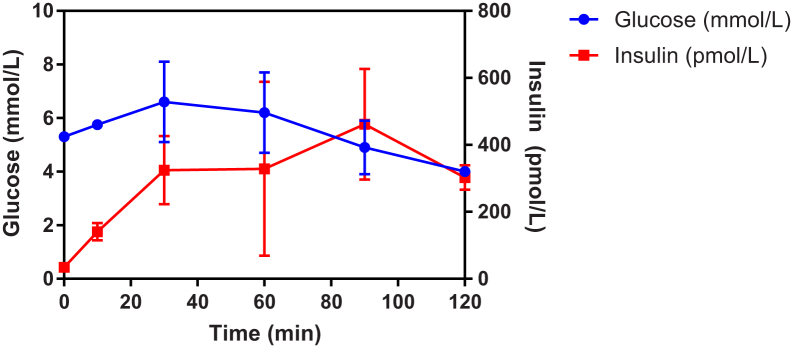
Assessment of glucose tolerance in patients with functional FD deficiency. Patient and sibling were given 75 g of oral glucose at 0 minutes and blood glucose was measured at regular intervals between 0 and 120 minutes. The *error bars* indicate the range of plasma glucose concentrations between the patient and sibling.

**Table E1:** Fasting measurements of metabolic parameters in the patient and sibling

Analyte	Patient	Sibling
Leptin (ng/mL)	10.6	11.3
Adiponectin (μg/mL)	9.7	6.5
NEFA (μmol/L)	391	212
Cholesterol (mmol/L)	4.2	4
HDL (mmol/L)	1.53	1.26
LDL (mmol/L)	2.3	2.3
Triglycerides (mmol/L)	0.8	0.9
HbA_1c_ (mmol/mol)	36	35

*HbA*_*1c*_, Glycosylated hemoglobin; *HDL*, high-density lipoprotein; *LDL*, low-density lipoprotein; *NEFA*, nonesterified fatty acids.

## Patient details

A 19-year-old, South Asian female presented with a 24-hour history of high fever, rigors, delirium, and diarrhea. On clinical examination, she was febrile with a purpuric rash and a reduced level of consciousness (Glasgow Coma scale score: 9 of 15). Intravenous antibiotic therapy was initiated for provisionally diagnosed meningococcal septicemia. She was intubated and transferred to the intensive care unit where she developed disseminated intravascular coagulation, for which she received treatment. Results from blood cultures drawn at the time of admission confirmed an infection with *Neisseria meningitides* serogroup Y. Her clinical condition improved with intensive care support and antimicrobial therapy. She was discharged after 2 weeks with minimal sequelae including bilateral leg scarring, a sacral pressure sore, and mild bilateral hearing loss.

At the age of 5 years, she had received bilateral tympanostomy tubes for recurrent ear infections and otitis media with effusion but had no other unusual infections as a child. She received the full course of childhood vaccinations as per the national immunization schedule.

On screening for immunodeficiency, laboratory measurement demonstrated a normal full blood count with normal counts of lymphoid cells. The titers of C3, C4, mannose-binding lectin and C1q were within normal range, but there was undetectable AP50 in conjunction with normal classical pathway hemolytic activity. In view of her complement deficiency, she was prescribed lifelong phenoxymethylpenicillin as antimicrobial prophylaxis. She was also vaccinated for meningitis serogroups A, C, W-135, and Y; meningitis C; pneumococcus; and *Haemophilus* influenza B to which she developed high antibody titer responses.

Her sole sibling, a younger male, who was homozygous for the same mutation, leading to an identical pattern on immunodeficiency screening, was healthy at assessment. He reported no excess of infections in the past. Of note, he reported having been treated empirically for suspected meningitis, at age 11 years, while travelling in Mauritius from which he recovered with no sequelae after a standard course of antibiotics.

## Functional FD deficiency does not result in impaired oral glucose tolerance

Recent preclinical evidence^E1^ that FD regulates insulin secretion prompted metabolic assessment of the patient and her sibling. They had a body mass index of 19.3 kg/m^2^ and 23.1 kg/m^2^, respectively. Fasting venous plasma glucose (5.2-5.4 mmol/L) and insulin (29-39 pmol/L) levels were normal in both subjects (Fig E3). Similarly, plasma glucose excursions were normal in response to an oral glucose (75 g) challenge. At 120 minutes following glucose administration, glucose levels remained normal (4.0 mmol/L). Furthermore, circulating concentrations of leptin and adiponectin, adipokines that regulate insulin sensitivity, were normal, as were fasting lipid profiles in both subjects. Thus, glucose homeostasis is not impaired in the context of genetic, and therefore lifelong, FD deficiency.

These results are consistent with the finding that FD knockout mice developed impaired glucose tolerance only on a long-term diabetogenic diet. This suggests that FD may contribute little to glucose homeostasis in the absence of prolonged metabolic stress. Alternatively, the role of FD in glucose homeostasis could be independent of binding to C3bB or independent of its serine protease activity and, by extension, independent of its downstream effects on the complement cascade. While congenital deficiency of FD alone may not lead to insulin insufficiency, Lo et al's^E1^ findings warrant observation of oral glucose tolerance in such FD-deficient patients under extreme metabolic stress and at older age. Further research will be required to understand the role of FD in glucose homeostasis and FD-deficient family pedigrees offer a useful clinical insight to this question.

## Methods

### AP50 measurement

AP100 RC003.1 Kit (Binding Site, Birmingham, UK) agar-chicken erythrocyte plates were prepared according to the manufacturer's instructions, with kit control and calibration solutions added. Then 5-μL aliquots of test serum were added to individual wells on the plates over ice. The loaded plates were then stored at 4°C for 18 hours to allow radial diffusion of serum components, followed by incubation at 37°C for 90 minutes to develop zones of lysis. The plates were then digitally scanned at high resolution, and the diameters of zones of lysis were measured using ImageJ 1.x computer software (National Institutes of Health, Bethesda, Md). Representative plates were selected for figures. The diameter of lysis correlates with AP50 and is expressed out of 100% relative to kit control. Purified human FD, FB, and properdin for reconstitution assays were purchased from Complement Tech (Tyler, Tex).

### Sanger sequencing

Genomic DNA was isolated from blood samples with QIAamp Kits (Qiagen, Hilden, Germany). The *CFD* gene PCR was performed with primers annealing to intron sequences close to each exon as described previously.^E2^ Specifically, regarding the R176P mutation, a 258-bp genomic fragment comprising exon 4 was amplified by PCR with the primers 5′-CTGGGGCATAGTCAACCAC-3′ and 5′-TGGGCCCTGTTCCTACTTG-3′. The cDNA numbering for the *CFD* variant identified is based on transcript Ref Seq accession no. NM_001928/Ensembl accession no. ENST00000327726.6 (National Center for Biotechnology Information, Bethesda, Md), beginning at the ATG start codon. The genomic coordinates refer to the GRCh37 genome build.

### Western blot analysis

Pooled control and the patient serum were diluted to 1:40 in Tris-buffered saline and resolved by SDS-PAGE on NuPAGE 4% to 12% Bis-Tris Gels, then blotted to nitrocellulose membranes. FD was detected using goat anti-human FD (AF1824; R&D Systems, Minneapolis, Minn) and donkey anti-goat-IgG IRDye 680CW (LI-COR Biosciences, Lincoln, Neb) secondary antibodies. The membranes were imaged using the Odyssey Infrared Imaging System (LI-COR Biosciences).

### Recombinant CFD expression and purification

Lentiviral transfer plasmid, envelope plasmid (pMD2.G; gift from Didier Trono; AddGene plasmid #12260; Cambridge, Mass) and packaging plasmid (psPAX2; AddGene; gift from Didier Trono; AddGene plasmid #12259) were used to transfect HEK293T cells to produce lentiviral particles. The transfer vector (modified pLenti-CMV-GFP-Puro; gift of Eric Campeau; AddGene plasmid #17448) included human FD cDNA (WT, R176P, R176A, S183A) with C-terminus hexahistidine tag upstream of an IRES-Thy1.1 and a puromycin resistance gene (Puro^R^). Transfection was carried out using Lipofectamine 3000 and, after 24 hours, the media containing the lentiviral particles was used immediately to stably transduce newly plated HEK293T cells. After puromycin selection, stably transduced 293T cells were incubated with FreeStyle media (Gibco, Thermo Fisher Scientific, Waltham, Mass) supplemented with 6X Glutamax and 2 mmol/L valproic acid. After 7 to 14 days, secreted recombinant CFD was purified from this media using cobalt immobilized metal affinity chromatography. CFD was eluted in 150 mmol/L imidazole in PBS and buffer exchanged by centrifugal concentration (Vivaspin 20; 10,000 Da pore size; Sartorius, Göttingen, Germany). Purity of the sample was confirmed on SDS-PAGE and mass spectrometry.

### Measuring *in vitro* catalytic activity of recombinant FD

Purified human C3b and FB were purchased from Complement Technology. Recombinant WT, R176P, or R176A FD were mixed in varying concentrations (1.0 μmol/L, 0.2 μmol/L, 0.04 μmol/L) with C3b (1.0 μmol/L) and FB (1.0 μmol/L) in veronal buffer (Lonza, Basel, Switzerland) with 10 mmol/L MgCl_2_ to a final volume of 20 μL. Reaction tubes were incubated for 10 minutes at 37°C before the addition of sample loading buffer (NuPAGE LDS Sample Buffer, Thermo Fisher Scientific) to terminate the reaction. The samples were then heated to 70°C for 10 minutes and resolved by SDS-PAGE on a Novex NuPAGE 4% to 12% Bis-Tris Gel. The gels were developed overnight with AcquaStain (Bulldog Bio, Portsmouth, NH), washed for 1 hour with distilled water, dried, and digitally scanned at high resolution. Analysis of percentage cleavage of FB was calculated by densitometry analysis using ImageJ 1.x computer software. Statistical comparisons between WT and R176P FD activity were performed at each concentration, from 4 independent experiments using the Kruskall-Wallis nonparametric *t* test.

### Circular dichroism spectroscopy and thermal shift assay

WT and R176P catalytically inactive (S183A) proteins were purified by size exclusion chromatography in chloride-free 0.1 mol/L sodium phosphate pH 7.0, diluted to a concentration of 2 mg/mL (72.4 μmol/L), and loaded into a 0.1-mm quartz sample cell. Circular dichroism spectra were recorded at 20°C on a Jasco J-810 spectropolarimeter (Oklahoma City, Okla) equipped with a Jasco PTC-348WI temperature controller. Spectra were acquired from 190 to 260 nm with 0.1-nm resolution and 1-nm bandwidth. Final spectra are the sum of 20 scans acquired at 50 nm/min. For the thermal shift assay, 2 μg of protein were mixed with SYPRO Orange (Fisher Scientific UK Ltd, Loughborough, UK) in PBS with 25 mmol/L HEPES and fluorescence data acquired on a ViiA 7 real-time PCR system with thermal denaturation over increasing temperatures observed using 1°C intervals.

### MD simulation of mutant FD

Starting models were derived from crystal structures of S183A FD (Protein Data Bank [PDB] ID: 2XW9, 1.2 Å resolution) reported previously.^E3^ The catalytic residue was reverted to serine during the MD setup. Coot^E4^ was used to place the Pro176 side chain in the Arg176 experimental density while minimizing clashes with surrounding atoms and aiming to achieve a favorable initial geometry. The resulting structures were further adjusted in UCSF Chimera (Resource for Biocomputing, Visualization, and Informatics, San Francisco, Calif).^E5^ The GROMACS package (Gromacs, University of Groningen, Groningen, The Netherlands)^E6^ was used to set up and run MD simulations. The AMBER99SB-ILDN force field (Gromacs)^E7^ and TIP3P water model were used and the structures placed in dodecahedral boxes with 10 Å padding and surrounded with solvent including water and 150 mmol/L NaCl. Following steepest gradient energy minimization, a modified Berendsen thermostat (2 groups, time constant 0.1 picoseconds, temperature 310 K) followed by a Berendsen barostat (isotropic, coupling constant 0.5 picoseconds, reference pressure 1 bar) were coupled to the system over 100 picoseconds. One hundred–nanosecond runs of unrestrained MD trajectories were produced. Following removal of periodic boundary condition artefacts, MD runs were visualized and analyzed in Chimera and bulk statistics extracted using GROMACS analysis routines.

### Surface plasmon resonance

Binding experiments were carried out based on established protocol using a Biacore T200 instrument.^E8^ FB and FD were buffer exchanged by gel filtration into veronal buffer with 10 mmol/L MgCl_2_. C3b was immobilized on the CM5 chip by amine coupling to achieve 8000 resonance units. A dual injection program was designed where 0.1 μmol/L or 1 μmol/L FB was injected at a flow rate of 30 μL/min for 3 minutes, followed by a second injection of a mix of 0.1 μmol/L or 1 μmol/L FB and FD at 30 μL/min for 4 minutes. After 5 minutes for dissociation, the chip was regenerated by 3 5-minute washes in 40 mmol/L acetate + 3 mol/L NaCl (pH 5.5). The chip was reequilibrated in assay buffer for 5 minutes. Catalytically inactive FD (S183A) or double mutant R176P/S183A were used to emulate the binding response of WT or R176P, respectively, while preventing cleavage of FB and subsequent dissociation of the complex.

### Esterolytic activity of FD

Z-Lys-SBzl was purchased from Sigma-Aldrich (St. Louis, Mo) in powder form and reconstituted to 100 mmol/L in 70% dimethyl sulfoxide. The assay buffer consisted of 50 mmol/L HEPES (pH 7.5), 220 mmol/L NaCl and 2 mmol/L of Ellman reagent (5,5-dithio-bis-[2-nitrobenzoic acid]; Sigma-Aldrich). Each reaction mixture contained FD (80 nmol/L), variable Z-Lys-SBzl concentrations (0.2-3.2 mmol/L) and 8% vol/vol of dimethyl sulfoxide in a final volume of 200 μL. Solutions were prewarmed to 37°C before addition of substrate to initiate the reaction. Hydrolysis of Z-lys-SBzl was measured using CLARIOstar FS microplate reader through equimolar formation of chromophore 2-nitro-5-thiobenzoate at 405 nm every 30 seconds for 90 minutes (ε = 13,600 mol/L^−1^cm^−1^). The rate of hydrolysis was determined from linear slopes of the reaction curves. Reaction velocities, expressed in apparent turnover values were plotted against substrate concentration.
